# The complete mitochondrial genome of *Euconocephalus Nasutus* and its phylogenetic analysis

**DOI:** 10.1080/23802359.2019.1662750

**Published:** 2019-09-09

**Authors:** Han Gao, Wei Tan, Xiaolei Yu, Weiling Jiang, Huanyu Zhang, Xiaoxuan Tian

**Affiliations:** Tianjin State Key Laboratory of Modern Chinese Medicine, Tianjin University of Traditional Chinese Medicine, Tianjin, China

**Keywords:** *Euconocephalus nasutus*, mitogenome, phylogeny

## Abstract

In this study, the first complete mitochondrial genome of genus *Euconocephalus* (*Euconocephalus nasutus*) was determined. The mitochondrial genome is 14,999 bp in length, including 13 protein-coding genes (PCGs), 22 transfer RNA genes (tRNAs), 2 ribosomal RNA genes, and an A + T-rich region. Phylogenetic analysis highly supported that *E. nasutus* showed a close relationship with *Ruspolia dubia.* The complete mitochondrial genome sequence of *E. nasutus* will provide fundamental data for the phylogenetic and biogeographic studies of the Tettigoniidae.

*Euconocephalus nasutus* belongs to family Tettigoniidae. In China, it is mainly distributed in the southwest region. *Euconocephalus nasutus* can be used for food and medicine (Wang 2012). Complete mitochondrial genome has been considered as a useful tool for population genetic and phylogenetic studies (Cameron 2014). Here, we report the complete mitochondrial genome sequence of *E. nasutus*, which is the first for the genus *Euconocephalus.* The annotated mitochondrial DNA sequence has been submitted to GenBank under accession number MN183101.

Adult specimens of *E. nasutus* were collected from Wanli (28.72 N, 115.74 E), Nanchang City, Jiangxi Province, China, on 18 April 2016. The sample was deposited at Tianjin State Key Laboratory of Modern Chinese Medicine (voucher number: bycm-1).

The mitochondrial genome of *E. nasutus* is 14,999 bp in length. The composition of the whole genome is 36.5% A, 34.7% T, 17.8% C, and 11.0% G. *E. nasutus* contains 13 protein-coding genes (PCGs), 22 transfer RNA genes (tRNAs), 2 ribosomal RNA genes, and an A + T-rich region. It is similar to the mitochondrial genome of same subfamily species *Ruspolia dubia* (Zhou et al. 2007).

The longest gene within this molecule is *ND5* containing 1732 bp and the shortest is *ATP8* gene which is 162 bp. Three PCGs initiation codons are ATT, six PCGs initiation codons are ATG, three PCGs initiation codons are ATA, except that COI starts with TCA. Correspondingly, most PCGs end with TAA, while COIII, ND5, CytB stop with T-Trna^Lys^, T-Trna^Phe^, TAG, respectively. MITOS Web Server and tRNAscan-SE (Lowe and Chan 2016) Search Server were employed to verify tRNAs based on their cloverleaf secondary structure.

To help us understand the phylogenetic position of *E. nasutus* in family Tettigoniidae, *E. nasutus* and other 11 published complete mitochondrial genome sequences in Tettigoniidae were used to reconstruct the maximum-likelihood (ML) phylogenetic tree (Figure 1). *Ommexecha virens* was served as outgroup. As expected, *E. nasutus* showed a close relationship with *Ruspolia dubia*, and further confirmed that *E. nasutus* belongs to the subfamily Conocephalinae. Moreover, the complete mitochondrial genome of *E. nasutus* can be used for further phylogenetic study within Tettigoniidae.

**Figure 1.**
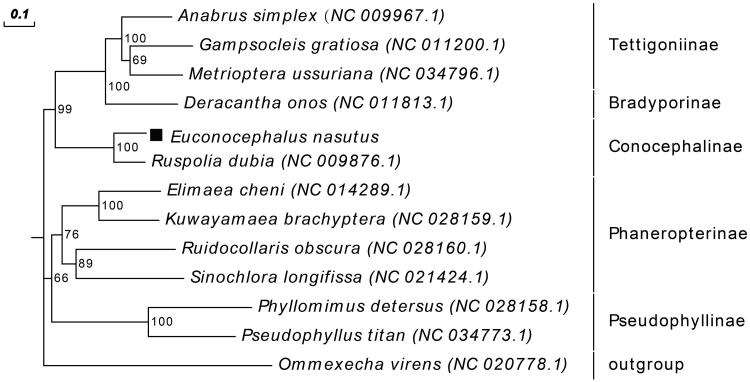
ML phylogenetic tree of *Euconocephalus nasutus* with 12 species was constructed by complete mitochondrial sequences. The numbers at each node indicate bootstrap support. GenBank accession numbers are given in brackets. Subfamilies of the sampled taxa are shown on the right. *Ommexecha virens* was selected as outgroup.

## References

[CIT0001] Cameron SL 2014 Insect mitochondrial genomics: implications for evolution and phylogeny. Annu Rev Entomol. 59(1):95–117. 24160435 10.1146/annurev-ento-011613-162007

[CIT0002] Lowe TM, Chan PP 2016 tRNAscan-SE on-line: integrating search and context for analysis of transfer RNA genes. Nucleic Acids Res. 44(W1):W54–57. 27174935 10.1093/nar/gkw413 PMC4987944

[CIT0003] Wang H 2012 Analysis and evaluation of the nutritional composition of *Euconocephalus nasutus* Thunberg. 49(5):1304–1308.

[CIT0004] Zhou Z, Huang Y, Shi F 2007 The mitochondrial genome of *Ruspolia dubia* (Orthoptera: Conocephalidae) contains a short A + T-rich region of 70 bp in length. Genome. 50(9):855–866. 17893726 10.1139/g07-057

